# MENTAL HEALTH OF GRANDPARENTS RAISING GRANDCHILDREN: UNDERSTANDING PREDICTORS OF GRANDPARENTS’ DEPRESSION

**DOI:** 10.1093/geroni/igz038.1042

**Published:** 2019-11-08

**Authors:** Youjung Lee, Kyeonghee Jang

**Affiliations:** 1 Binghamton University, Binghamton, New York, United States; 2 Abilene Christian University, Abilene, Texas, United States

## Abstract

Grandparents raising grandchildren experience caregiving stress, negatively influencing their mental health. They experience limited social supports and suffer from a lack of respite care and community resources. The present study attempts to explore needs of grandparent-headed families and factors related to grandparents’ depression. In 2015-19, surveys with 92 custodial grandparents were conducted in the northeastern U.S. The respondents were primarily white (77%) and 62 years old on average (ranged from 44 through 84) at the time of the interviews. The depressive symptoms ranged from 1 through 45, with the score of 16+ indicating clinical depression (41%). Sixty-three percent of grandparents reported a household income below $40,000 and 40% of them rated their health as poor or fair. Ninety-five percent reported at least one or more concerns in raising their grandchild (i.e., financial concerns, legal issues, and physical health). A multiple linear regression analysis was performed to examine the contributions of age, ethnicity, duration of care, factors related to multigenerational caregiving, social support from family members, social support from friends, and social support from significant others in accounting for grandparents’ depressive symptoms. The model explained 29% of variance in the outcome (R2=.290; adjusted R2=.231). Among the predictors, only one factor was significant: social support from family members (beta=-.352, p=.006). Grandparents with increased social support from family members have lower rates of depression. This finding reinforces the importance of familial support for grandparents raising grandchildren, and recommends the development of family-centered programs to offer support for custodial grandparents to promote caregivers’ well-being.

